# Differential Effects of Outpatient Portal User Status on Inpatient Portal Use: Observational Study

**DOI:** 10.2196/23866

**Published:** 2021-04-30

**Authors:** Naleef Fareed, Pallavi Jonnalagadda, Sarah R MacEwan, Gennaro Di Tosto, Seth Scarborough, Timothy R Huerta, Ann Scheck McAlearney

**Affiliations:** 1 The Center for the Advancement of Team Science, Analytics, and Systems Thinking College of Medicine The Ohio State University Columbus, OH United States; 2 Department of Biomedical Informatics College of Medicine The Ohio State University Columbus, OH United States; 3 Department of Family and Community Medicine College of Medicine The Ohio State University Columbus, OH United States

**Keywords:** patient portals, technology experience, health information technology, technology acceptance, technology use, log files, mobile phone

## Abstract

**Background:**

The decision to use patient portals can be influenced by multiple factors, including individuals’ perceptions of the tool, which are based on both their personal skills and experiences. Prior experience with one type of portal may make individuals more comfortable with using newer portal technologies. Experienced outpatient portal users in particular may have confidence in their ability to use inpatient portals that have similar functionality. In practice, the use of both outpatient and inpatient portal technologies can provide patients with continuity of access to their health information across care settings, but the influence of one type of portal use on the use of other portals has not been studied.

**Objective:**

This study aims to understand how patients’ use of an inpatient portal is influenced by outpatient portal use.

**Methods:**

This study included patients from an academic medical center who were provided access to an inpatient portal during their hospital stays between 2016 and 2018 (N=1571). We analyzed inpatient portal log files to investigate how inpatient portal use varied by using 3 categories of outpatient portal users: prior users, new users, and nonusers.

**Results:**

Compared with prior users (695/1571, 44.24%) of an outpatient portal, new users (214/1571, 13.62%) had higher use of a select set of inpatient portal functions (messaging function: incidence rate ratio [IRR] 1.33, 95% CI 1.06-1.67; function that provides access to the outpatient portal through the inpatient portal: IRR 1.34, 95% CI 1.13-1.58). Nonusers (662/1571, 42.14%), compared with prior users, had lower overall inpatient portal use (all active functions: IRR 0.68, 95% CI 0.60-0.78) and lower use of specific functions, which included the function to review vitals and laboratory results (IRR 0.51, 95% CI 0.36-0.73) and the function to access the outpatient portal (IRR 0.53, 95% CI 0.45-0.62). In comparison with prior users, nonusers also had lower odds of being comprehensive users (defined as using 8 or more unique portal functions; odds ratio [OR] 0.57, 95% CI 0.45-0.73) or composite users (defined as comprehensive users who initiated a 75th or greater percentile of portal sessions) of the inpatient portal (OR 0.42, 95% CI 0.29-0.60).

**Conclusions:**

Patients’ use of an inpatient portal during their hospital stay appeared to be influenced by a combination of factors, including prior outpatient portal use. For new users, hospitalization itself, a major event that can motivate behavioral changes, may have influenced portal use. In contrast, nonusers might have lower self-efficacy in their ability to use technology to manage their health, contributing to their lower portal use. Understanding the relationship between the use of outpatient and inpatient portals can help direct targeted implementation strategies that encourage individuals to use these tools to better manage their health across care settings.

## Introduction

### Background

Health information technologies (HITs) are increasingly being introduced and implemented to provide individuals with tools to help them manage their health. Patient portals are one such HIT tool that provides patients with the opportunity to learn about their health and participate in their health care [1]. These portals offer patients access to personal health information and educational materials, a means to manage their health and health care, and methods to communicate with their health care providers. There is evidence that links the use of patient portals to better patient-provider communication, clinical decision making, and patient satisfaction [2,3]. Furthermore, the use of patient portals has been associated with improved health outcomes such as controlled blood pressure and better glycemic and cholesterol control in patients with chronic diseases [4,5].

Despite the potential benefits of patient portals, there are still barriers to their adoption and use. First, individuals must have access to the internet to use outpatient portals [6]. In addition, individuals must have adequate eHealth literacy—defined as the ability to acquire, comprehend, and apply health information from electronic sources [7,8]—to understand the information that a patient portal can provide [9]. The decision to use patient portals also involves multiple factors related to an individual’s perception of the tool in the context of their personal skills and experiences. For example, prior experience with technology may allow individuals to become more comfortable with new technologies compared with individuals who do not have technology experience [10]. Prior technology experience may be particularly valuable in supporting the adoption of technologies that are similar, as this experience may give individuals confidence in their abilities to use other technologies with similar functionalities [11].

Patient portals were first employed in outpatient settings but are now increasingly being implemented in inpatient settings, with functionality tailored to the hospital environment [12]. Inpatient portals have been suggested to have multiple benefits for patients, including the promotion of independence, reduction of anxiety, and increasing empowerment of patients during their hospitalization [13]. Their use has been shown to help uncover medical errors, improve medication adherence, and facilitate patient-provider communication [14]. Furthermore, there is evidence supporting the association between inpatient portal use and lower hospital readmission rates [15], medical errors, and adverse drug events [16]. In practice, the use of both outpatient and inpatient portals together can provide patients with continuity of access to their health information and their health care providers across care settings [17], but the influence of one type of portal use on the use of the other has not been studied.

### Objectives

This study seeks to understand the relationship between the use of an outpatient portal and the use of an inpatient portal by examining the portal use of 3 groups of patients: patients who had used an outpatient portal before using an inpatient portal during hospitalization (*prior users*); patients who used an outpatient portal only after using an inpatient portal during a hospital stay (*new users*); and patients who did not use an outpatient portal, despite the fact that they used an inpatient portal during their hospitalization (*nonusers*). We hypothesized that prior users of the outpatient portal would have higher frequency of use and more comprehensive use of the inpatient portal compared with new users or nonusers of the outpatient portal and that these differences would persist after adjusting for demographic and clinical variables.

## Methods

### Study Setting and Design

A large-scale, pragmatic, randomized controlled trial (RCT) was conducted across 6 noncancer hospitals at a large academic medical center (AMC) between September 2016 and August 2019 [18]. The trial considered the use of 2 patient portals developed by Epic Systems (Epic Systems Corporation). The inpatient portal application, MyChart Bedside (MCB), available on tablet computers provisioned as part of the hospital admission, was introduced in 2013 in select units of the AMC and rolled out system wide in 2016. The outpatient portal, MyChart (MC), was a web-based patient portal that had been in use at the AMC since 2011 and was available as an app on a variety of personal devices, including computers and cell phones. The overall objective of the RCT was to examine how the introduction of MCB influenced patient experience. Briefly, the RCT was structured to examine the impact of 2 interventions, technology and training, using a factorial design. Individuals were provided with a tablet with either full-tech (full MCB) functionality or lite-tech (limited MCB) functionality and received either a high-touch (in-person training from a technology navigator to use the patient portal) or low-touch (instructional video) intervention. In the context of this larger study, we examined MCB use in relation to MC user status among participants in the arm of the RCT that was not impacted by study design considerations—that is, patients who were provisioned with the full-tech tablets and received the low-touch intervention.

### MCB and MC Functions

The MCB app available to hospitalized patients included the following functions: Access MyChart (log in to or create an MC account), Dining on Demand (order food from a predefined menu), Happening Soon (view expected interactions with care team during the hospital stay), I Would Like (request ancillary services), Messages (communicate with the care team), My Health (review vitals and laboratory results), Notes (type notes), Taking Care of Me (view active members of the care team), To Learn (access educational materials), and Tutorial (view a tutorial on how to use MCB). The Access MyChart function could be used by inpatients to create an MC account that was available in the outpatient environment. We refer to all these functions together as active functions in contrast to the administrative functions that were not part of our investigation. The methods for processing MCB log files have been previously described [19].

The MC functions included Messaging (links to messaging center, letters to the patient, and prescription refill option), Visits (list of past and upcoming visits), My Record (list of medications and allergies, medical history and immunizations, test results and health summary, preventive care, and a summary of plan of care), Medical Tools (share medical records with other services, participate in research studies, and connect tracking devices), Resources (terms and conditions, patient education, and frequently asked questions), Proxy (request or renew proxy), and Preferences (personal and security settings and notification preferences). Although MC is tailored for outpatient care, it also includes access to information from inpatient stays within 3 days of discharge. The methods for processing MC log files have also been described previously [20].

### Study Data

We used audit log file data from September 2016 to August 2018, a period after MCB rollout that was considered stable with respect to MCB implementation, to test associations between MCB use by MC user status. Our analysis was restricted to a subsample who received access to the full suite of MCB functions and an instructional training video available on the tablet. This subsample provided an adequate sample size to test associations between MCB use by MC user status and avoided confounding effects that might have been related to the in-person technology navigation intervention. Our analytic sample comprised 1571 patients who met the study criteria for this focused analysis.

Our primary outcomes were based on the frequency of MCB function use. We defined MCB function use at 3 levels: the patient level, representing use aggregated over multiple admissions; the admission level, representing use during a single admission; and the sessions level, representing a single, defined instance of coherent MCB use. The definition of MCB use metrics has been previously described in detail [19]. In short, MCB use was defined as the number of times a function was used. We calculated the frequency of use of each of the 10 MCB functions as well as the total frequency of use of all 10 MCB functions.

First, we determined the number of MCB sessions for each patient. Next, we aggregated MCB function use from up to the first 3 admissions for all patients, where the first admission was when patients were enrolled into the RCT (ie, enrollment admission). We selected this cutoff for the number of admissions as most patients had admissions that fell within this range (number of admissions; median 1; 90th percentile=3). We excluded patients whose length of stay (LOS) during their enrollment admission was less than 3 days and those who had no recorded MCB use during their enrollment admission. We also calculated the comprehensiveness of MCB use, defined as the use of 8 or more unique MCB functions [19]. Finally, we defined composite use, a combination of comprehensive use and high-frequency use of MCB. High-frequency MCB use was defined as the total number of MCB sessions equal to or greater than the 75th percentile. We report the results at the patient level to present the aggregate perspective of patient use.

Our main predictor variable was MC user status at the time MCB use first occurred, which we defined as having occurred upon enrollment into the RCT. To define MC user status, we selected 90 days as the cutoff point based on the pattern of MC account activations among patients after their enrollment into the RCT. An MC account is activated when a user signs up to use the app. The number of MC account activations plateaued 3 months after enrollment, which was the basis for our decision that MC account activations occurring within 90 days of trial enrollment was a reasonable time frame during which portal activations could be considered. MC use was defined as any use of the MC functions. Using these definitions, patients were classified into 3 groups: (1) *prior users* (695/1571, 44.24%) were patients with any past recorded MC use before their enrollment admission, (2) *new users* (214/1571, 13.62%) were patients who first used MC during their enrollment admission or within 90 days of their enrollment into the RCT, and (3) *nonusers* (662/1571, 42.14%) were patients who did not use MC before, during, or within 90 days after enrollment. Other covariates included age at enrollment, sex, race, the length of time (days) the tablet with MCB was provisioned (ie, length of provisioning [LOP]) to the patient during their admission, and the Charlson Comorbidity Index (CCI). We did not include the LOS as a covariate because of variability in the length of time between admission and provisioning of the tablet to the patient; given this variability, the LOS does not reflect the duration of time that the patient had to use the inpatient tablet with MCB. Of note, this intersection between the LOS and LOP has been previously described in work pertaining to the processing of log files [19].

### Data Analysis

For our analysis, we first examined the distributions of the outcome and predictor variables and assessed differences in the demographic characteristics of MC user groups via analysis of variance, Chi-square tests, and Kruskal-Wallis tests, as applicable. We then modeled the frequency of MCB use by using logistic and negative binomial regression and adjusting for demographic variables, the LOP, and CCIs.

We also performed several sensitivity analyses. First, we repeated the process of calculating MCB function use at the admission and sessions levels from up to 3 admissions. The purpose of examining MCB function use at different levels was to gain a better understanding of patient engagement with the app by considering both granular (sessions) and more aggregate (admission and patient) data. The 1571 patients in our sample had 2227 admissions and 53,823 sessions. Of the 2227 admissions, 1025 (46.02%) were prior users, 310 (13.92%) were new users, and 892 (40.05%) were nonusers. Of the 53,823 sessions, 25,810 (47.95%) were initiated by prior users, 9481 (17.62%) by new users, and 18,532 (34.43%) by nonusers. Our estimates for SEs were clustered by patient. We did not define comprehensiveness and composite use at the sessions level, as the recording of function use may be influenced by system idiosyncrasies, such as automatically logging the patient out after 10 minutes of inactivity [19-21]. Second, we restricted the analysis to MCB function use to the first admission or the enrollment admission. Finally, we repeated the analyses by redefining MC user status as follows: (1) *prior users* (n=695) were patients with any past recorded MC use before their enrollment admission; (2) *new users* (n=240) were patients who first used MC during their enrollment admission or within 180 days of their enrollment into the trial; and (3) *nonusers* (n=636) were patients who did not use MC before, during, or within 180 days after their enrollment in to the RCT.

## Results

### Baseline Characteristics of the Participants

Table 1 summarizes the characteristics of the study participants for the analytic sample based on MC user status. The sample mostly consisted of female (899/1571, 57.22%) and White (1180/1571, 75.11%) participants, and participants had a mean age of 47 years (SD 15.09). The median CCI was 1 (range 0-15), and the median LOP was 7 (range 1-124) days. Across the different MC user groups, there were statistically significant differences in gender, race, age, and CCIs. Prior users were typically older, female, and White. New users were likely to have the portal provisioned to them for one more day than the other user groups.

The frequency of MCB use in relation to MC user status is summarized in Table 2. The prior users group had a median of 23 MCB sessions per patient, whereas new users and nonusers had 29 and 16 sessions per patient, respectively. Among prior users, 34.9% (243/695) were comprehensive users and 19.4% (135/695) were composite users. Among new users, 36.9% (79/214) were comprehensive users and 23.4% (50/214) were composite users. For nonusers, 23.1% (153/662) were classified as comprehensive and 9.8% (65/662) were classified as composite users.

**Table 1:** Baseline characteristics of the study population by MyChart user status.

Characteristics			Overall (N=1571)		Prior users (n=695)		New users (n=214)		Nonusers (n=662)		*P* value^a^	
Gender (female), n (%)			899 (57.22)		433 (62.30)		127 (59.35)		339 (51.21)		<.001	
**Race, n (%)**												.004
	Black	326 (20.75)		119 (17.12)		42 (19.63)		165 (24.92)				
	White	1180 (75.11)		541 (77.84)		162 (75.70)		477 (72.05)				
	Other	65 (4.14)		35 (5.04)		10 (4.67)		20 (3.02)				
Age (years), mean (SD)			47 (15.09)		48 (14.95)		44 (14.75)		46 (15.15)		<.001	
Charlson Comorbidity Index, median (range)			1 (0-15)		1 (0-15)		1 (0-11)		1 (0-13)		<.001	
Length of provisioning (days), median (range)			7 (1-124)		7 (1-124)		8 (1-116)		7 (2-117)		.05	

^a^Differences in gender and race were examined using the Chi-square test. Analysis of variance was used to assess differences in mean age. The Kruskal-Wallis test was used to assess differences in the Charlson Comorbidity Index and length of provisioning.

**Table 2:** Frequency of MyChart Bedside use by MyChart user status^a^.

Characteristics	Overall (N=1571)	Prior users (n=695)	New users (n=214)	Nonusers (n=662)
Number of sessions, median (Q1^b^, Q3^c^)	20 (10, 41)	23 (12, 44)	29 (14, 51)	16 (8, 31)
Active functions, median (Q1, Q3)	93 (37, 198)	103 (44, 237)	136 (58, 259)	69 (26, 151)
Access MyChart, median (Q1, Q3)	2 (0, 4)	2 (1, 5)	4 (1, 7)	1 (0, 2)
Dining on Demand, median (Q1, Q3)	14 (4, 31)	15 (6, 33)	20 (6, 40)	9 (3, 26)
Happening Soon, median (Q1, Q3)	24 (6, 76)	27 (7, 82)	45 (14, 98)	17 (3, 61)
I Would Like, median (Q1, Q3)	0 (0, 0)	0 (0, 0)	0 (0, 0)	0 (0, 0)
Messages, median (Q1, Q3)	2 (0, 5)	2 (1, 6)	3 (1, 7)	1 (0, 3)
My Health, median (Q1, Q3)	1 (0, 16)	3 (0, 22)	5 (0, 27)	0 (0, 4)
Notes, median (Q1, Q3)	0 (0, 0)	0 (0, 0)	0 (0, 0)	0 (0, 0)
Taking Care of Me, median (Q1, Q3)	2 (1, 6)	3 (1, 8)	4 (1, 10)	2 (0, 5)
To Learn, median (Q1, Q3)	0 (0, 1)	0 (0, 1)	0 (0, 2)	0 (0, 1)
Tutorial, median (Q1, Q3)	6 (4, 11)	7 (4, 12)	7 (5, 13)	6 (3, 9)
Comprehensive user^d^, n (%)	475 (30.24)	243 (34.96)	79 (36.92)	153 (23.11)
Composite user^e^, n (%)	250 (15.91)	135 (19.42)	50 (23.36)	65 (9.81)

^a^MyChart (MC) user status was defined as follows: prior users (n=695) were patients with any past recorded MC use before their enrollment admission; new users (n=214) were patients who first used MC during their enrollment admission or within 90 days of their enrollment into the trial; and nonusers (n=662) were patients who did not use MC either before, during, or after their enrollment admission or those who first used MC 90 days after enrollment into the trial.

^b^Q1: 25th percentile.

^c^Q3: 75th percentile.

^d^A comprehensive user was defined as those who used 8 or more MyChart Bedside functions at both the patient and admission levels.

^e^A composite user was defined as a comprehensive user and high-frequency user of MyChart Bedside. High-frequency users were defined as users who had a total number of MyChart Bedside sessions greater than or equal to the 75th percentile (41 sessions).

### Association Between MCB Use and MC User Status

The association between MCB use and MC user status is summarized in Table 3. In the unadjusted analysis, with the exception of the Notes and I Would Like functions, there were significant differences across the 3 MC user groups in the use of the remaining 8 MCB functions. Overall, compared with prior users, we found that new users had higher use of MCB, and nonusers had lower use of MCB. After adjustment, although the frequency of use of active MCB functions was not significantly different between new users and prior users, we found a significantly higher use of certain individual MCB functions among new users. For instance, new users had 33% higher use of the Messages function (incidence rate ratio [IRR] 1.33, 95% CI 1.06-1.67; *P*=.02) and 34% higher use of the Access MyChart function (IRR 1.34, 95% CI 1.13-1.58; *P*=.001), compared with prior users. Furthermore, new users had 12% higher use of the Tutorial function (IRR 1.12, 95% CI 0.99-1.26; *P*=.07), compared with prior users. In contrast, nonusers had a significantly lower frequency of MCB use compared with prior users for all but 2 functions (ie, I Would Like and Notes), even after adjusting for demographic factors, the LOP, and CCIs. The full model estimates are available in Multimedia Appendix 1.

**Table 3:** MyChart Bedside function use in relation to MyChart user status (in reference to prior users) at the patient level^a^.

Functions			New users (unadjusted)		New users (adjusted^b^)		Nonusers (unadjusted)		Nonusers (adjusted^b^)
**Number of sessions**									
	IRR^c^ (95% CI)	1.20 (0.99-1.46)		1.07 (0.93-1.23)		0.75 (0.65-0.86)		0.74 (0.67-0.82)	
	*P* value	.07		.35		<.001		<.001	
**Active functions**									
	IRR (95% CI)	1.24 (0.98-1.56)		1.11 (0.93-1.32)		0.69 (0.58-0.82)		0.69 (0.60-0.79)	
	*P* value	.07		.27		<.001		<.001	
**Access MyChart**									
	IRR (95% CI)	1.38 (1.16-1.67)		1.34 (1.13-1.58)		0.54 (0.46-0.64)		0.53 (0.45-0.62)	
	*P* value	<.001		.001		<.001		<.001	
**Dining on Demand**									
	IRR (95% CI)	1.20 (1.01-1.44)		1.10 (0.96-1.27)		0.81 (0.69-0.95)		0.79 (0.70-0.88)	
	*P* value	.04		.16		.01		<.001	
**Happening Soon**									
	IRR (95% CI)	1.38 (1.00-1.92)		1.16 (0.90-1.50)		0.71 (0.56-0.91)		0.68 (0.56-0.82)	
	*P* value	.05		.26		.01		<.001	
**I Would Like**									
	IRR (95% CI)	1.59 (0.89-2.84)		1.47 (0.83-2.61)		0.96 (0.67-1.37)		0.97 (0.68-1.38)	
	*P* value	.12		.19		.84		.87	
**Messages**									
	IRR (95% CI)	1.45 (1.15-1.83)		1.33 (1.06-1.67)		0.67 (0.55-0.83)		0.65 (0.53-0.78)	
	*P* value	.002		.02		<.001		<.001	
**My Health**									
	IRR (95% CI)	0.89 (0.55-1.42)		0.93 (0.66-1.32)		0.44 (0.30-0.65)		0.51 (0.36-0.73)	
	*P* value	.61		.69		<.001		<.001	
**Notes**									
	IRR (95% CI)	0.81 (0.30-2.23)		0.63 (0.24-1.71)		0.58 (0.25-1.32)		0.54 (0.24-1.24)	
	*P* value	.69		.36		.19		.15	
**Taking Care of Me**									
	IRR (95% CI)	1.22 (0.99-1.51)		1.09 (0.90-1.33)		0.65 (0.54-0.79)		0.64 (0.54-0.75)	
	*P* value	.07		.35		<.001		<.001	
**To Learn**									
	IRR (95% CI)	1.18 (0.84-1.64)		1.14 (0.81-1.59)		0.74 (0.58-0.95)		0.75 (0.58-0.95)	
	*P* value	.34		.45		.02		.02	
**Tutorial**									
	IRR (95% CI)	1.11 (0.99-1.25)		1.12 (0.99-1.26)		0.89 (0.81-0.98)		0.92 (0.84-1.00)	
	*P* value	.10		.07		.02		.05	
**Comprehensive user^d^**									
	OR^e^ (95% CI)	1.09 (0.79-1.50)		1.02 (0.73-1.42)		0.56 (0.44-0.71)		0.57 (0.45-0.73)	
	*P* value	.60		.90		<.001		<.001	
**Composite user^f^**									
	OR (95% CI)	1.26 (0.88-1.83)		1.15 (0.77-1.72)		0.45 (0.33-0.62)		0.42 (0.29-0.60)	
	*P* value	.21		.50		<.001		<.001	

^a^MyChart (MC) user status was defined as follows: prior users (n=695) were patients with any past recorded MC use before their enrollment admission; new users (n=214) were patients who first used MC during their enrollment admission or within 90 days of their enrollment into the trial; and nonusers (n=662) were patients who did not use MC either before, during, or after their enrollment admission or those who first used MC 90 days after enrollment into the trial.

^b^Adjusted for age at enrollment, female gender, race, Charlson Comorbidity Index, and the length of provisioning time of the inpatient tablet.

^c^IRR: incidence rate ratio.

^d^A comprehensive user was defined as those who used 8 or more MyChart Bedside functions at both the patient and admission levels.

^e^OR: odds ratio.

^f^A composite user was defined as a comprehensive user and high-frequency user of MyChart Bedside (MCB). High-frequency users were defined as users who had a total number of MCB sessions greater than or equal to the 75th percentile (41 sessions).

### Association Between Comprehensive and Composite Use by MC User Status

Table 3 also summarizes the association between MCB comprehensive use and composite use by MC user status. Prior users and new users were similar in terms of comprehensive and composite MCB use. However, compared with prior users, nonusers had lower odds of being comprehensive users (odds ratio [OR] 0.57, 95% CI 0.45-0.73; *P*<.001) or composite users (OR 0.42, 95% CI 0.29-0.60; *P*<.001).

### Sensitivity Analyses of the Association Between MCB Use and MC User Status

Multimedia Appendices 2 and 3 summarize the findings from our sensitivity analyses at the admission and sessions levels. These results were largely consistent with those from analyses conducted at the patient level. There were notable exceptions at the admission level: the Dining on Demand function was used 12% more among new users (patient level: IRR 1.10, 95% CI 0.96-1.27; *P*=.16; admission level: IRR 1.12, 95% CI 0.98-1.28, *P*=.10) compared with prior users and the use of the Tutorial function was not significantly different between nonusers and prior users (patient level: IRR 0.92, 95% CI 0.84-1.00; *P*=.05; admission level: IRR 0.99, 95% CI 0.91-1.08, *P*=.85). At the sessions level, the use of the Dining on Demand (IRR 1.02, 95% CI 0.98-1.06; *P*=.28) and Tutorial functions (IRR 1.04, 95% CI 0.97-1.12; *P*=.27) were similar between prior users and new users. For the Access MyChart (patient: IRR 1.34, 95% CI 1.13-1.58; *P*=.001; admission: IRR 1.37, 95% CI 1.16-1.61; *P*<.001; session: IRR 1.25, 95% CI 1.15-1.36; *P*<.001) and Messages (patient: IRR 1.33, 95% CI 1.06-1.67; *P*=.02; admission: IRR 1.37, 95% CI 1.09-1.71; *P*=.01; session: IRR 1.24, 95% CI 1.14-1.34; *P*<.001) functions, we found smaller differences between new and prior users than at the patient and admission levels.

Although nonusers had lower use of MCB compared with prior users, the difference between the 2 groups was smaller than that found at the patient and admission levels. At the sessions level, the frequency of use of the Happening Soon, Messages, and To Learn functions was not statistically different between nonusers and prior users. In contrast to the patient and admission levels, where nonusers had significantly lower use of most MCB functions compared with prior users, at the sessions level, the use of the Tutorial and Dining on Demand functions was with notable exceptions. Compared with prior users, nonusers had 27% higher use of the Tutorial function (IRR 1.27, 95% CI 1.20-1.32; *P*<.001) and 9% higher use of the Dining on Demand function (IRR 1.09, 95% CI 1.06-1.12; *P*<.001). Although the I Would Like function did not differ by MC user status at the other 2 levels, at the sessions level, nonusers had 38% higher use (IRR 1.38, 95% CI 0.99-1.91; *P*=.06) compared with prior users.

Multimedia Appendix 4 summarizes the results from our sensitivity analyses using information solely from the enrollment admission into the RCT (model 2) and those using information from up to 3 admissions but also using 180 days after enrollment as the cutoff to define MC user status (model 3). For comparison, we provide the results from the analysis of MCB use from up to 3 admissions and 90 days after enrollment (the cutoff to define MC user status; model 1). On restricting the analysis to the enrollment admission, the results were generally consistent with our main findings, with some exceptions. New users had 19% higher use (IRR 1.19, 95% CI 1.00-1.42; *P*=.048) of all active MCB functions compared with prior users. Furthermore, compared with prior users, new users had 15% (IRR 1.15, 95% CI 1.00-1.32; *P*=.05) and 30% (IRR 1.30, 95% CI 1.02-1.67; *P*=.04) higher use of the Dining on Demand and Happening Soon functions, respectively. Finally, compared with prior users, new users had 44% (OR 1.44, 95% CI 1.12-1.85; *P*=.01) greater odds of being comprehensive users of MCB. Using 180 days after enrollment in the trial as the cutoff to define MC user status did not affect the number of prior users (n=695), but there were more new users (n=240) and fewer nonusers (n=636). Our findings were consistent with the results of analyses using 90 days after enrollment in the trial as the cutoff to define MC user status. 

## Discussion

### Principal Findings

In our study of inpatient portal use in relation to outpatient portal user status, we found that the use of specific portal functions as well as the frequency and comprehensiveness of inpatient portal use differed across user groups. Our analyses showed that Dining on Demand and Happening Soon were the most frequently used inpatient portal functions, whereas I Would Like and Notes were the least frequently used functions. These results align with previous findings from a cluster analysis, which also highlighted the variability in function use based on distinct behaviors of subgroups [21]. Among new users in this study, we found more use of the Tutorial, Messages, and Access MyChart functions than those among prior users. Nonusers were found to use most MCB functions less than prior users. Nonusers were also less likely to be comprehensive or composite users of MCB than prior users.

Prior research has found that an individual’s experience with technology may increase their adoption of new technologies by increasing their familiarity with technology and reducing their dependence on external resources for help [22]. Experience may thereby impact an individual’s perceptions of the usefulness and ease of use of a new technology [23,24]. This may, in part, explain our finding of higher inpatient portal use by prior users compared with nonusers. At the same time, individuals with less technology experience may use technologies differently (ie, use fewer functions or use less frequently) than those with more technology experience [25], and this prior finding may explain the differences we observed in portal function use between prior users and nonusers. As previous studies have shown that individuals with less prior technology experience may benefit from supplemental education or ongoing support to help them use new technologies [10], it is possible that training patients without prior experience of portal functionality or the benefits of using the portal may increase portal use for nonusers.

Prior users in our sample were mostly older, female, and White. This is similar to the results of another recent study on portal usage from 3 iterations of the Health Information National Trends Survey, which showed that portal users were more likely to be female, White, and have higher education or income. Furthermore, this prior study also showed a widening gender gap and narrowing age gap in portal use among Americans over time [26].

Beyond experience with the outpatient portal, there are likely additional factors that influence both patients’ use of the inpatient portal while hospitalized and their use of the outpatient portal post discharge. For instance, barriers to portal use such as low levels of patient activation or low health literacy may contribute to a patient’s choice not to use patient portals; such factors may have been at play among the nonusers in our study. Low patient activation may diminish the patient’s interest in using portals, as it has been shown that individuals with lower levels of activation have a lower likelihood of using the internet to access health information [27]. In addition, low health literacy has been recognized as a barrier to the use of patient portals, as this may prevent patients from effectively comprehending information found via the portal [28].

The abovementioned barriers to portal adoption and use are more likely to be experienced by vulnerable and underserved patients [29,30], and it is concerning that these patients may also be left behind in the use of portals and in leveraging the potential benefits these tools provide [2-5,14]. Notably, the digital divide may also explain some differences in MCB use among nonusers. Although the inpatient portal is offered on hospital-provided tablets with wireless internet connectivity, the use of the outpatient portal requires a patient to have access to both technology and the internet. Not all individuals have this access, contributing to the digital divide that has been found [31]. For instance, households with low incomes (<US $30,000 per year) are less likely to have home broadband internet access, computers, or smartphones [32]. In addition, enrollment to use an outpatient portal and use of its secure messaging function have been shown to be associated with whether individuals have internet access in their home neighborhoods [6]. Individuals may be less likely to adopt and use an inpatient portal when they do not have internet access at home to enable the use of an outpatient portal post discharge. It is also likely that nonusers include patients visiting the health system for a specific procedure but whose usual care is provided through a separate health system that has a different patient portal platform. These individuals may be less motivated to use either MCB or MC if they perceive their interaction with the AMC to be only temporary.

Interestingly, we found greater use of the inpatient portal for a specific set of MCB functions, such as the Messages function, among new users compared with prior users. As hospitalization may represent a major life event that may spark behavioral changes and increase an individual’s engagement in their health care [33], this experience may have contributed to new users’ high levels of MCB use during their hospital stay and their creation of MC accounts to manage their health after discharge. New users may also have used the Messages function more because of its novelty and the high expectations placed on it by this highly motivated group of users. In contrast, prior users may have viewed the Messages function as potentially less useful, and more research is needed to understand what factors may have contributed to this behavior.

However, we did not find differences between the new user and prior user groups in the odds of being a composite or comprehensive user. Although these findings contradict our study hypothesis, these results suggest that it is possible that prior users may have had a priori assumptions that they already completely understand portal functionality and perhaps overlooked unique functions that were available in the inpatient portal. Promoting awareness of patient portals and their varied functions may be important across settings, as it can support seamless care transitions and maintain patient engagement. Ultimately, knowing more about the relationship between the use of outpatient and inpatient portals can help inform implementation practices that encourage individuals to use these tools across care settings to better manage their health and participate in their health care.

The results at the patient level were generally consistent with those at the admission and sessions levels. However, there were some notable exceptions. New users had higher numbers of sessions compared with prior users when analyzed at the sessions level (Multimedia Appendices 2 and 3 provide detailed results at the admission and sessions levels). Nonusers had significantly higher use of the Dining on Demand, I Would Like, and Tutorial functions. Furthermore, at the sessions level, there were no significant differences in the use of the Messages and To Learn functions between nonusers and prior users. The results at the other levels, especially at the sessions level, could be highly sensitive to additional system artifacts in spite of our processing of the raw log file data, and more research is required to explore how different data processing techniques and analytical models may account for these potential idiosyncrasies.

Finally, our findings were generally consistent when examined using information on MCB function use solely from the enrollment admission. It is notable that new users had significantly higher use of all active functions in the enrollment admission, but this association was no longer significant when examining MCB use from up to 3 admissions. This pattern was observed for the Happening Soon and Dining on Demand functions, suggesting a novelty effect. Similarly, the odds of being a comprehensive user were high when examining MCB use from enrollment admission alone.

### Limitations

Limitations should be considered when interpreting our results. First, our analysis focused on a single health system and a single patient portal platform, potentially restricting the generalizability of our findings. However, our inclusion of multiple hospitals across this health system and the similarities in patient portal functionality across this and other vendor platforms does support the potential applicability of our findings to other settings. Second, we restricted our focus to the associations between outpatient portal use and inpatient portal use, without considering the alternative influence of inpatient portal use on outpatient portal use. Although we decided to examine this relationship because outpatient portals are more established than inpatient portals, giving patients more opportunity to have been introduced to outpatient portal technology, future studies can be designed to study the alternate relationship. Third, although experience with technology can be measured in multiple ways [34], we defined experience as having an active outpatient portal account. Additional work examining more granular measures such as length of experience with patient portals might provide greater insight into the impact of experience on patient portal use. Fourth, MC users can access the same set of MC functions using the inpatient tablet or personal devices. Therefore, the difference in MCB use (specifically, number of sessions, active functions, and Access MyChart function) by MC user status may be influenced by a patient’s use of their mobile device to access their MC app during an inpatient stay. Finally, our study was not designed to explore the implications of portal use on health outcomes. Although functions such as My Health and To Learn have been previously noted to influence patients’ health outcomes [21], studying such relationships within and among outpatient user groups was beyond the scope of this study.

### Conclusions

Together, outpatient and inpatient portals can provide patients access to their health information and a means to communicate with their health care providers, including along the continuum of care. We found that in comparison with patients who had previously used the outpatient portal, new users of the outpatient portal had higher inpatient portal use for a select set of functions, whereas nonusers of the outpatient portal had lower use of the inpatient portal during their hospitalization. Understanding how the use of one type of patient portal affects the use of the other can help us better understand how these tools can be both implemented and promoted to increase patients’ involvement in their health care across settings.

## Appendix

Multimedia Appendix 1 Fully adjusted models showing MyChart Bedside function use in relation to MyChart user status (in reference to prior users and White patients) at the patient level. These models used information from up to 3 admissions, including the study enrollment admission.

Multimedia Appendix 2 Frequency of MyChart Bedside function use in relation to MyChart user status at the patient, admission, and sessions levels from up to 3 admissions, including the study enrollment admission.

Multimedia Appendix 3 MyChart Bedside function use in relation to MyChart user status at the patient, admission, and sessions levels.

Multimedia Appendix 4 MyChart Bedside function use in relation to MyChart user status at the patient level among different samples (in reference to prior users).

## Abbreviations

AMC: academic medical center
CCI: Charlson Comorbidity Index
HIT: health information technology
IRR: incidence rate ratio
LOP: length of provisioning
LOS: length of stay
MC: MyChart
MCB: MyChart Bedside
OR: odds ratio
RCT: randomized controlled trial
