# Supplementary material for: Differential Effects of Outpatient Portal User Status on Inpatient Portal Use: Observational Study
Source: J Med Internet Res. 2021 Apr 30;23(4):e23866. doi: 10.2196/23866 (PMC8122294; doi:10.2196/23866)
Supplement: Multimedia Appendix 1 [file jmir_v23i4e23866_app1.docx]

**Differential effects of outpatient portal user status on inpatient portal use: Observational study**

Multimedia Appendix 1

# **Multimedia Appendix 1. Fully-adjusted models showing MyChart Bedside function use in relation to MyChart user status (in reference to Prior Users and White race) at the patient level using information from up to three admissions, inclusive of the study enrollment admission**

|  | **New Users** | **Non-Users** | **Age** | **Female** | **Black** | **Other race** | **LOP** | **CCI** |
| --- | --- | --- | --- | --- | --- | --- | --- | --- |
| Number of sessions  IRR (95% CI) | 1.07  (0.93, 1.23)  *P*=0.35 | 0.74  (0.67, 0.82)  *P*<.001 | 0.99  (0.99, 099)  *P*<.001 | 1.01  (0.91, 1.11)  *P*=0.91 | 0.79  (0.70, 0.88)  *P*<.001 | 0.88  (0.70, 1.10)  *P*=0.26 | 1.05  (1.04, 1.06)  *P*<.001 | 0.98  (0.96, 1.01)  *P*=0.15 |
| Active Tasks  IRR (95% CI) | 1.11  (0.93, 1.32) *P*=0.27 | 0.69  (0.60, 0.79)  *P*<.001 | 0.99  (0.98, 0.99)  *P*<.001 | 0.99  (0.87, 1.12)  *P*=0.85 | 0.77  (0.66, 0.90)  *P*=0.001 | 0.81  (0.63, 1.05)  *P*=0.12 | 1.05  (1.04, 1.06)  *P*<.001 | 0.99  (0.96, 1.02)  *P*=0.59 |
| Access MyChart  IRR (95% CI) | 1.34  (1.13, 1.58)  *P*=0.001 | 0.53  (0.45, 0.62)  *P*<.001 | 0.99  (0.98, 0.99)  *P*<.001 | 1.01  (0.88, 1.17)  *P*=0.84 | 0.68  (0.57, 0.80)  *P*<.001 | 0.88  (0.61, 1.28)  *P*=0.50 | 1.02  (1.02, 1.03)  *P*<.001 | 1.01  (0.98, 1.05)  *P*=0.39 |
| Dining on Demand  IRR (95% CI) | 1.10  (0.96, 1.27)  *P*=0.16 | 0.79  (0.70, 0.88)  *P*<.001 | 0.99  (0.99, 1.00)  *P*<.001 | 1.15  (1.04, 1.28)  *P*=0.01 | 0.84  (0.74, 0.95)  *P*=0.006 | 0.92  (0.72, 1.18)  *P*=0.50 | 1.06  (1.05, 1.06)  *P*<.001 | 0.99  (0.96, 1.01)  *P*=0.33 |
| Happening Soon  IRR (95% CI) | 1.16  (0.90, 1.50)  *P*=0.26 | 0.68  (0.56, 0.82)  *P*<.001 | 0.98  (0.98, 0.99)  *P*<.001 | 0.95  (0.79, 1.13)  *P*=0.55 | 0.77  (0.63, 0.95)  *P*=0.01 | 0.75  (0.51, 1.10)  *P*=0.14 | 1.05  (1.04, 1.07)  *P*<.001 | 0.99  (0.95, 1.03)  *P*=0.64 |
| I Would Like  IRR (95% CI) | 1.47  (0.83, 2.61)  *P*=0.19 | 0.97  (0.68, 1.38)  *P*=0.87 | 0.99  (0.98, 1.01)  *P*=0.23 | 1.12  (0.79, 1.59)  *P*=0.52 | 0.90  (0.56, 1.44)  *P*=0.66 | 1.11  (0.50, 2.46)  *P*=0.79 | 1.03  (1.02, 1.05)  *P*<.001 | 1.02  (0.95, 1.10)  *P*=0.51 |
| Messages  IRR (95% CI) | 1.33  (1.06, 1.67)  *P*=0.02 | 0.65  (0.53, 0.78)  *P*<.001 | 0.98  (0.98, 0.99)  *P*<.001 | 1.03  (0.86, 1.22)  *P*=0.77 | 0.84  (0.67, 1.05)  *P*=0.12 | 0.89  (0.59, 1.35)  *P*=0.59 | 1.03  (1.02, 1.04)  *P*<.001 | 1.01  (0.97, 1.05)  *P*=0.58 |
| My Health  IRR (95% CI) | 0.93  (0.66, 1.32)  *P*=0.69 | 0.51  (0.36, 0.73)  *P*<.001 | 0.99  (0.98, 1.00)  *P*=0.11 | 0.92  (0.68, 1.26)  *P*=0.61 | 0.58  (0.37, 0.91)  *P*=0.02 | 0.70  (0.38, 1.28)  *P*=0.25 | 1.05  (1.03, 1.07)  *P*<.001 | 1.00  (0.94, 1.07)  *P*=0.94 |
| Notes  IRR (95% CI) | 0.63  (0.23, 1.71)  *P*=0.36 | 0.54  (0.24, 1.24)  *P*=0.15 | 0.97  (0.94, 0.99)  *P*=0.02 | 2.29  (1.02, 5.15)  *P*=0.05 | 0.57  (0.27, 1.22)  *P*=0.15 | 1.23  (0.33, 4.63)  *P*=0.76 | 0.99  (0.97, 1.02)  *P*=0.52 | 1.06  (0.90, 1.24)  *P*=0.52 |
| Taking Care of Me  IRR (95% CI) | 1.09  (0.90, 1.33)  *P*=0.35 | 0.64  (0.54, 0.75)  *P*<.001 | 0.99  (0.98, 0.99)  *P*<.001 | 1.02  (0.98, 0.99)  *P*=0.80 | 0.75  (0.61, 0.94)  *P*=0.01 | 0.86  (0.61 ,1.22)  *P*=0.39 | 1.04  (1.03, 1.05)  *P*<.001 | 0.99  (0.95, 1.05)  *P*=0.44 |
| To Learn  IRR (95% CI) | 1.14  (0.81, 1.59)  *P*=0.45 | 0.75  (0.58, 0.95)  *P*=0.02 | 1.00  (0.99, 1.01)  *P*=0.72 | 1.25  (0.99, 1.58)  *P*=0.06 | 1.36  (1.01, 1.85)  *P*=0.05 | 0.91  (0.59, 1.42)  *P*=0.69 | 1.03  (1.02, 1.04)  *P*<.001 | 1.00  (0.95, 1.06)  *P*=0.99 |
| Tutorial  IRR (95% CI) | 1.12  (0.99, 1.26)  *P*=0.07 | 0.92  (0.84, 1.00)  *P*=0.05 | 1.01  (1.00, 1.01)  *P*<.001 | 1.03  (0.95, 1.11)  *P*=0.52 | 0.94  (0.85, 1.04)  *P*=0.22 | 1.10  (0.92, 1.32)  *P*=0.28 | 1.02  (1.01, 1.02)  *P*<.001 | 1.00  (0.98, 1.01)  *P*=0.28 |
| Comprehensive user^a^  OR (95% CI) | 1.02  (0.73, 1.42)  *P*=0.90 | 0.57  (0.45, 0.73)  *P*<.001 | 0.99  (0.99, 1.00)  *P*=0.12 | 1.41  (1.12, 1.78)  *P*=0.003 | 0.76  (0.57, 1.01)  *P*=0.06 | 0.87  (0.49, 1.55)  *P*=0.64 | 1.03  (1.02, 1.04)  *P*<.001 | 0.99  (0.94, 1.04)  *P*=0.64 |
| Composite user^b^  OR (95% CI) | 1.15  (0.77, 1.72)  *P*=0.50 | 0.42  (0.29, 0.60)  *P*<.001 | 0.99  (0.98, 1.00)  *P*=0.04 | 1.37  (1.00, 1.88)  *P*=0.05 | 0.60  (0.40, 0.90)  *P*=0.01 | 0.51  (0.21, 1.27)  *P*=0.15 | 1.05  (1.04, 1.07)  *P*<.001 | 1.01  (0.94, 1.09)  *P*=0.74 |

MyChart user status defined as Prior Users (n=695) were patients with any past recorded MC use prior to their enrollment admission; New Users (n=214) who first used MC during their enrollment admission or within 90 days of their enrollment into the trial; and Non-Users (n=662) who did not use MC either before, during, or after their enrollment admission or those who first used MC 90 days after enrollment into the trial.

^a^ Comprehensive user defined as use of eight or more MCB functions at patient level.

^b^ Composite user defined as a comprehensive user and high-frequency user of MCB defined as having total number of MCB sessions greater than or equal the 75^th^ percentile (41 sessions).

IRR, incidence rate ratio; OR, odds ratio; CI, confidence interval; LOP, length of provisioning time of inpatient tablet; CCI, Charlson Comorbidity Index.
