# Supplementary material for: Differential Effects of Outpatient Portal User Status on Inpatient Portal Use: Observational Study
Source: J Med Internet Res. 2021 Apr 30;23(4):e23866. doi: 10.2196/23866 (PMC8122294; doi:10.2196/23866)
Supplement: Multimedia Appendix 2 [file jmir_v23i4e23866_app2.docx]

**Differential effects of outpatient portal user status on inpatient portal use: Observational study**

Multimedia Appendix 2

# **Multimedia Appendix 2. Frequency of MyChart Bedside function use in relation to MyChart user status at the patient, admission, and sessions levels with up to three admissions, inclusive of the study enrollment admission**

|  | **Overall** | **Prior Users** | **New Users** | **Non-Users** |
| --- | --- | --- | --- | --- |
| Number of sessions  Median (min, max)  Patient^a^  Admission^b^ | 20 (1, 484)  15 (1, 385) | 23 (1, 436)  16 (1, 385) | 29 (2, 484)  18 (1, 325) | 16 (1, 344)  12 (1, 290) |
| Active Functions  Median (min, max)  Patient  Admission  Sessions^c^ | 93 (1, 3,310)  63 (1, 2,127)  3 (1, 224) | 103 (2, 3,310)  72 (1, 1,885)  3 (1, 140) | 136 (4, 2,924)  89 (1, 2,127)  3 (1, 172) | 69 (1,1,983)  49 (1, 1,911)  5 (1, 224) |
| Access MyChart  Median (min, max)  Patient  Admission  Sessions | 2 (0, 56)  1 (0, 56)  0 (0, 4) | 2 (0, 37)  1 (0, 31)  0 (0, 3) | 4 (0, 37)  2 (0, 29)  0 (0, 4) | 1 (1, 56)  0 (0, 56)  0 (0, 4) |
| Dining on Demand  Median (min, max)  Patient  Admission  Sessions | 14 (0, 378)  10 (0, 329)  0 (0, 32) | 15 (0, 378)  11 (0, 329)  0 (0, 25) | 20 (0, 199)  13 (0, 199)  0 (0, 19) | 9 (0, 283)  8 (0, 195)  1 (0, 32) |
| Happening Soon  Median (min, max)  Patient  Admission  Sessions | 24 (0, 2,447)  16 (0, 1,595)  0 (0, 223) | 27 (0, 2,447)  17 (0, 1,248)  0 (0, 139) | 45 (0, 2,093)  25 (0, 1,595)  0 (0, 171) | 17 (0, 1,451)  11 (0, 1,451)  0 (0, 223) |
| I Would Like  Median (min, max)  Patient  Admission  Sessions | 0 (0, 19)  0 (0, 15)  0 (0, 8) | 0 (0, 10)  0 (0, 8)  0 (0, 5) | 0 (0, 19)  0 (0, 15)  0 (0, 6) | 0 (0, 10)  0 (0, 10)  0 (0, 8) |
| Messages  Median (min, max)  Patient  Admission  Sessions | 2 (0, 125)  1 (0, 112)  0 (0, 22) | 2 (0, 125)  1 (0, 112)  0 (0, 11) | 3 (0, 58)  2 (0, 48)  0 (0, 12) | 1 (0, 82)  1 (0, 60)  0 (0, 22) |
| My Health  Median (min, max)  Patient  Admission  Sessions | 1 (0, 1883)  0 (0, 862)  0 (0, 112) | 3 (0, 1883)  1 (0, 862)  0 (0, 112) | 5 (0, 1053)  3 (0, 769)  0 (0, 68) | 0 (0, 977)  0 (0, 754)  0 (0, 98) |
| Notes  Median (min, max)  Patient  Admission  Sessions | 0 (0, 17)  0 (0, 12)  0 (0, 5) | 0 (0, 17)  0 (0, 12)  0 (0, 5) | 0 (0, 5)  0 (0, 5)  0 (0, 4) | 0 (0, 7)  0 (0, 7)  0 (0, 5) |
| Taking Care of Me  Median (min, max)  Patient  Admission  Sessions | 2 (0, 93)  2 (0, 92)  0 (0, 10) | 3 (0, 92)  2 (0, 92)  0 (0, 10) | 4 (0, 60)  2 (0, 51)  0 (0, 8) | 2 (0, 93)  1 (0, 65)  0 (0, 10) |
| To Learn  Median (min, max)  Patient  Admission  Sessions | 0 (0, 48)  0 (0, 31)  0 (0, 28) | 0 (0, 48)  0 (0, 31)  0 (0, 28) | 0 (0, 26)  0 (0, 22)  0 (0, 18) | 0 (0, 20)  0 (0, 17)  0 (0, 14) |
| Tutorial  Median (min, max)  Patient  Admission  Sessions | 6 (0, 94)  5 (0, 63)  0 (0, 21) | 7 (0, 62)  5 (0, 45)  0 (0, 21) | 7 (0, 43)  5 (0, 39)  0 (0, 10) | 6 (0, 94)  4 (0, 63)  0 (0, 14) |
| Comprehensive user^d^  n (%)  Patient  Admission | 30 (475)  21 (477) | 35 (243)  23 (240) | 37 (79)  27 (83) | 23 (153)  17 (154) |
| Composite user^e^  n (%)  Patient  Admission | 16 (250)  12 (276) | 19 (135)  14 (140) | 23 (50)  18 (55) | 10 (65)  9 (81) |

^a^Among the 1,571 patients, 44% (695) were Prior Users, 14% (214) were New Users, and 42% (662) were Non-Users.

^b^1,571 patients had 2,227 admissions from up to three admissions inclusive of enrollment admission; of the 2,227 admissions, 46% (1,025) were Prior Users, 14% (310) were New Users, and 40% (892) were Non-Users.

^c^1,571 patients had 53,823 sessions from up to three admissions inclusive of enrollment admission; of the 53,823 sessions, 48% (25,810) were Prior Users, 18% (9,481) were New Users, and 34% (18,532) were Non-MCA users.

^d^Comprehensive user defined as use of eight or more MCB functions at patient and admission levels.

^e^Composite user at the patient level defined as a comprehensive user and high-frequency user of MCB, defined as having total number of MCB sessions greater than or equal the 75^th^ percentile (41 sessions); composite user at the admission level defined as a comprehensive user and high-frequency user of MCB, defined as having total number of MCB sessions greater than or equal the 75^th^ percentile (29 sessions).
