# Supplementary material for: Differential Effects of Outpatient Portal User Status on Inpatient Portal Use: Observational Study
Source: J Med Internet Res. 2021 Apr 30;23(4):e23866. doi: 10.2196/23866 (PMC8122294; doi:10.2196/23866)
Supplement: Multimedia Appendix 3 [file jmir_v23i4e23866_app3.docx]

**Differential effects of outpatient portal user status on inpatient portal use: Observational study**

Multimedia Appendix 3

# **Multimedia Appendix 3: MyChart Bedside function use in relation to MyChart user status at the patient, admission, and sessions levels**

| **MyChart Bedside**  **functions** | **Level of analysis** | **New Users**  **unadjusted** | **New Users**  **adjusted** | **Non-Users**  **unadjusted** | **Non-Users**  **adjusted** |
| --- | --- | --- | --- | --- | --- |
| Number of sessions  IRR (95% CI) | Patient^a^ | 1.20 (0.99, 1.46)  *P*=0.07 | 1.07 (0.93, 1.23)  *P*=0.3 | 0.75(0.65, 0.86)  *P* < .001 | 0.74 (0.67, 0.82)  *P* < .001 |
|  | Admission^b^ | 1.23 (1.02, 1.47)  *P*=0.03 | 1.09 (0.94, 1.26)  *P*=0.25 | 0.82 (0.71, 0.94)  *P*=0.004 | 0.77 (0.69, 0.86)  *P* < .001 |
| Active Functions  IRR (95% CI) | Patient | 1.24 (0.98, 1.56)  *P*=0.07 | 1.11 (0.93, 1.32)  *P*=0.27 | 0.69 (0.58, 0.82)  *P* < .001 | 0.69 (0.60, 0.79)  *P* < .001 |
|  | Admission | 1.26 (1.01, 1.57)  *P*=0.04 | 1.13 (0.93, 1.37)  *P*=0.23) | 0.75 (0.64, 0.89)  *P* =0.001 | 0.70 (0.60, 0.80)  *P* < .001 |
|  | Sessions^c^ | 1.04 (0.93, 1.17)  *P*=0.44 | 1.04 (1.00, 1.08)  *P*=0.08 | 0.89 (0.81, 0.99)  *P*=0.03 | 0.89 (0.86, 0.92)  *P*< .001 |
| Access MyChart  IRR (95% CI) | Patient | 1.38 (1.16, 1.67)  *P* < .001 | 1.34 (1.13, 1.58)  *P* =0.001 | 0.54 (0.46, 0.64)  *P* < .001 | 0.53 (0.45, 0.62)  *P* < .001 |
|  | Admission | 1.41 (1.19, 1.67)  *P* < .001 | 1.37 (1.16, 1.61)  *P* < .001 | 0.59 (0.50, 0.70)  *P* < .001 | 0.58 (0.49, 0.68)  *P* < .001 |
|  | Sessions | 1.16 (0.97,1.39)  *P*=0.10 | 1.25 (1.15, 1.36)  *P* < .001 | 0.72 (0.61, 0.84)  *P* < .001 | 0.75 (0.70, 0.81)  *P* < .001 |
| Dining on Demand  IRR (95% CI) | Patient | 1.20 (1.01, 1.44)  *P*=0.042 | 1.10 (0.96, 1.27)  *P*=0.16 | 0.81 (0.69, 0.95)  *P*=0.01 | 0.79 (0.70, 0.88)  *P* < .001 |
|  | Admission | 1.23 (1.04, 1.45)  *P*=0.02 | 1.12 (0.98, 1.28)  *P*=0.10 | 0.89 (0.77, 1.03)  *P*=0.11 | 0.83 (0.74, 0.92)  *P* =0.001 |
|  | Sessions | 1.01 (0.90, 1.14)  *P*=0.89 | 1.02 (0.98, 1.06)  *P*=0.28 | 1.08 (0.99, 1.17)  *P*=0.08 | 1.09 (1.06, 1.12)  *P* < .001 |
| Happening Soon  IRR (95% CI) | Patient | 1.38 (1.00, 1.92)  *P*=0.052 | 1.16 (0.90, 1.50)  *P*=0.26 | 0.71(0.56, 0.91)  *P*=0.01 | 0.68 (0.56, 0.82)  *P* < .001 |
|  | Admission | 1.41 (1.03, 1.93)  *P*=0.03 | 1.21 (0.90, 1.62)  *P*=0.21 | 0.78 (0.62, 0.99)  *P*=0.043 | 0.67 (0.55, 0.83)  *P* < .001 |
|  | Sessions | 1.16 (0.95, 1.41)  *P*=0.14 | 1.11 (1.05, 1.17)  *P* < .001 | 0.95 (0.81, 1.11)  *P*=0.50 | 0.92 (0.88, 0.96)  *P* < .001 |
| I Would Like  IRR (95% CI) | Patient | 1.59 (0.89, 2.84)  *P*=0.12 | 1.47 (0.83, 2.61)  *P*=0.19 | 0.96 (0.67, 1.37)  *P*=0.84 | 0.97 (0.68, 1.38)  *P*=0.87 |
|  | Admission | 1.62 (0.92, 2.87)  *P*=0.10 | 1.53 (0.85, 2.74)  *P*=0.15 | 1.05 (0.74, 1.50)  *P*=0.77 | 1.05 (0.74, 1.47)  *P*=0.80 |
|  | Sessions | 1.33 (0.76, 2.35)  *P*=0.32 | 1.38 (0.99, 1.91)  *P*=0.06 | 1.28 (0.90, 1.82)  *P*=0.17 | 1.33 (01.00, 1.77)  *P*=0.05 |
| Messages  IRR (95% CI) | Patient | 1.45 (1.15, 1.83)  *P*=0.002 | 1.33 (1.06, 1.67)  *P*=0.02 | 0.67 (0.55, 0.83)  *P* < .001 | 0.65 (0.53, 0.78)  *P* < .001 |
|  | Admission | 1.48 (1.18, 1.85)  *P* =0.001 | 1.37 (1.09, 1.71)  *P*=0.01 | 0.74 (0.61, 0.90)  *P* =0.003 | 0.70 (0.58, 0.86)  *P* < .001 |
|  | Sessions | 1.22 (0.98, 1.50)  *P*=0.07 | 1.24 (1.14, 1.34)  *P* < .001 | 0.90 (0.75, 1.07)  *P*=0.22 | 0.91 (0.85, 0.98)  *P*=0.13 |
| My Health  IRR (95% CI) | Patient | 0.89 (0.55, 1.42)  *P*=0.61 | 0.93 (0.66, 1.32)  *P*=0.69 | 0.44 (0.30, 0.65)  *P* < .001 | 0.51 (0.36, 0.73)  *P* < .001 |
|  | Admission | 0.90 (0.57, 1.43)  *P*=0.66 | 0.90 (0.63, 1.29)  *P*=0.56 | 0.49 (0.33, 0.71)  *P* < .001 | 0.49 (0.35, 0.70)  *P* < .001 |
|  | Sessions | 0.74 (0.49, 1.13)  *P*=0.17 | 0.79 (0.70, 0.89)  *P* < .001 | 0.59 (0.41, 0.84)  *P*=0.004 | 0.60 (0.54, 0.67)  *P* < .001 |
| Notes  IRR (95% CI) | Patient | 0.81 (0.30, 2.23)  *P*=0.69 | 0.63 (0.24, 1.71)  *P*=0.36 | 0.58 (0.25, 1.32)  *P*=0.19 | 0.54 (0.24, 1.24)  *P*=0.15 |
|  | Admission | 0.83 (0.30, 2.25)  *P*=0.71 | 0.65 (0.25, 1.66)  *P*=0.36 | 0.63 (0.28, 1.44)  *P*=0.28 | 0.67 (0.29, 1.54)  *P*=0.35 |
|  | Sessions | 0.68 (0.25, 1.83)  *P*=0.45 | 0.70 (0.30, 1.61)  *P*=0.40 | 0.77 (0.34, 1.74)  *P*=0.52 | 0.75 (0.47, 1.20)  *P*=0.26 |
| Taking Care of Me  IRR (95% CI) | Patient | 1.22 (0.99, 1.51)  *P*=0.07 | 1.09 (0.90, 1.33)  *P*=0.35 | 0.65 (0.54, 0.79)  *P* < .001 | 0.64 (0.54, 0.75)  *P* < .001 |
|  | Admission | 1.24 (1.01, 1.52)  *P*=0.04 | 1.12 (0.92, 1.35)  *P*=0.25 | 0.71 (0.59, 0.85)  *P* < .001 | 0.68 (0.58, 0.81)  *P* < .001 |
|  | Sessions | 1.02 (0.88, 1.18)  *P*=0.77 | 1.05 (0.98, 1.12)  *P*=0.19 | 0.86 (0.75, 0.99)  *P*=0.04 | 0.88 (0.83, 0.93)  *P* < .001 |
| To Learn  IRR (95% CI) | Patient | 1.18 (0.84, 1.64)  *P*=0.34 | 1.14 (0.81, 1.59)  *P*=0.45 | 0.74 (0.58, 0.95)  *P*=0.02 | 0.75 (0.58, 0.95)  *P*=0.02 |
|  | Admission | 1.20 (0.86, 1.66)  *P*=0.28 | 1.18 (0.84, 1.67)  *P*=0.33 | 0.81 (0.64, 1.04)  *P*=0.09 | 0.81 (0.63, 1.03)  *P*=0.09 |
|  | Sessions | 0.99 (0.70, 1.39)  *P*=0.93 | 1.11 (0.85, 1.45)  *P*=0.45 | 0.99 (0.76, 1.26)  *P*=0.91 | 1.04 (0.85, 1.27)  *P*=0.70 |
| Tutorial  IRR (95% CI) | Patient | 1.11 (0.99, 1.25)  *P*=0.102 | 1.12 (0.99, 1.26)  *P*=0.07 | 0.89 (0.81, 0.98)  *P*=0.02 | 0.92 (0.84,1.00)  *P*=0.05 |
|  | Admission | 1.13 (0.99, 1.28)  *P*=0.06 | 1.12 (0.99, 1.27)  *P*=0.08 | 0.98 (0.89, 1.07)  *P*=0.61 | 0.99 (0.91, 1.08)  *P*=0.85 |
|  | Sessions | 0.93 (0.79, 1.10)  *P*=0.38 | 1.04 (0.97, 1.12)  *P*=0.27 | 1.18 (1.05, 1.33)  *P*=0.01 | 1.27 (1.20, 1.32)  *P* < .001 |
| Comprehensive user^d^  OR (95% CI) | Patient | 1.09 (0.79, 1.50)  *P*=0.60 | 1.02 (0.73, 1.42)  *P*=0.90 | 0.56 (0.44, 0.71)  *P* < .001 | 0.57 (0.45, 0.73)  *P* < .001 |
|  | Admission | 1.20 (0.89, 1.61)  *P*=24 | 1.12 (0.83, 1.52)  *P*=0.45 | 0.68 (0.54, 0.87)  *P*=0.002 | 0.68 (0.53, 0.86)  *P*=0.002 |
| Composite user^e^  OR (95% CI) | Patient | 1.26 (0.88, 1.83)  *P*=0.21 | 1.15 (0.77, 1.72)  *P*=0.50 | 0.45 (0.33, 0.62)  *P* < .001 | 0.42(0.29, 0.60)  *P* < .001 |
|  | Admission | 1.36 (0.97, 1.92)  *P*=0.08 | 1.24 (0.86, 1.78)  *P*=0.25 | 0.63 (0.47, 0.85)  *P*=0.003 | 0.61 (0.44, 0.82)  *P*=0.001 |

Models adjusted for age at enrollment, female gender, race, Charlson Comorbidity Index, and length of provisioning time of the inpatient tablet. Adjusted model reference Prior Users, White, Male.

^a^ Among the 1571 patients, 44% (695) were Prior Users, 14% (214) were New Users, and 42% (662) were Non-Users.

^b^ 1,571 patients had 2,227 admissions from up to three admissions inclusive of enrollment admission; of the 2,227 admissions, 46% (1,025) were Prior Users, 14% (310) were New Users, and 40% (892) were Non-Users.

^c^ 1,571 patients had 53,823 sessions from up to three admissions inclusive of enrollment admission; of the 53,823 sessions, 48% (25,810) were Prior Users, 18% (9,481) were New Users, and 34% (18,532) were Non-Users.

^d^ Comprehensive user defined as use of eight or more MCB functions at both patient and admission levels.

^e^ Composite user at the patient level defined as a comprehensive user and high-frequency user of MCB, defined as having total number of MCB sessions greater than or equal the 75^th^ percentile (41 sessions); composite user at the admission level defined as a comprehensive user and high-frequency user of MCB defined as having total number of MCB sessions greater than or equal the 75^th^ percentile (29 sessions).

IRR, incidence rate ratio; OR, odds ratio; CI, confidence interval.
