# Supplementary material for: Differential Effects of Outpatient Portal User Status on Inpatient Portal Use: Observational Study
Source: J Med Internet Res. 2021 Apr 30;23(4):e23866. doi: 10.2196/23866 (PMC8122294; doi:10.2196/23866)
Supplement: Multimedia Appendix 4 [file jmir_v23i4e23866_app4.docx]

**Differential effects of outpatient portal user status on inpatient portal use: Observational study**

Multimedia Appendix 4

# **Multimedia Appendix 4. MyChart Bedside function use in relation to MyChart user status at the patient level using different samples (in reference to Prior Users)**

| **MyChart Bedside functions** |  | **New Users**  **Unadjusted** | **New Users**  **adjusted** | **Non-Users**  **unadjusted** | **Non-Users**  **adjusted** |
| --- | --- | --- | --- | --- | --- |
| Number of sessions  IRR (95% CI) | Model 1^a^ | 1.20 (0.99, 1.46)  *P*=0.07 | 1.07 (0.93, 1.23)  *P*=0.35 | 0.75(0.65, 0.86)  *P* < .001 | 0.74 (0.67, 0.82)  *P* < .001 |
|  | Model 2^b^ | 1.27* (1.06, 1.52)  *P*=0.01 | 1.11 (0.97, 1.27)  *P*=0.12 | 0.80 (0.70, 0.92)  *P*=0.001 | 0.77 (0.69, 0.85)  *P* < .001 |
|  | Model 3^c^ | 1.16 (0.96, 1.40)  *P*=0.12 | 1.04 (0.91, 1.20)  *P*=0.55 | 0.74 (0.64, 0.86)  *P* < .001 | 0.74 (0.66, 0.82)  *P* < .001 |
| Active Functions  IRR (95% CI) | Model 1 | 1.24 (0.98, 1.56)  *P*=0.07 | 1.11 (0.93, 1.32)  *P*=0.27 | 0.69 (0.58, 0.82)  *P* < .001 | 0.69 (0.60, 0.79)  *P* < .001 |
|  | Model 2 | 1.35 (1.09, 1.67)  *P*=0.01 | 1.19 (1.00, 1.42)  *P*=0.048 | 0.78 (0.66, 0.92)  *P*=0.004 | 0.73(0.64, 0.84)  *P* < .001 |
|  | Model 3 | 1.18 (0.95, 1.48)  *P*=0.14 | 1.07 (0.90, 1.31)  *P*=0.44 | 0.69 (0.57, 0.82)  *P* < .001 | 0.68(0.60, 0.78)  *P* < .001 |
| Access MyChart  IRR (95% CI) | Model 1 | 1.38 (1.16, 1.67)  *P* < .001 | 1.34 (1.13, 1.58)  *P* =0.001 | 0.54 (0.46, 0.64)  *P* < .001 | 0.53 (0.45, 0.62)  *P* < .001 |
|  | Model 2 | 1.35 (1.12, 1.60)  *P* < .001 | 1.31 (1.12, 1.55)  *P*=0.001 | 0.52(0.45, 0.61)  *P* < .001 | 0.51(0.44, 0.59)  *P* < .001 |
|  | Model 3 | 1.33 (1.11, 1.57)  *P*=0.001 | 1.28 (1.09, 1.51)  *P*=0.003 | 0.53 (0.44, 0.63)  *P* < .001 | 0.52 (0.44, 0.61)  *P* < .001 |
| Dining on Demand  IRR (95% CI) | Model 1 | 1.20 (1.01, 1.44)  *P*=0.042 | 1.10 (0.96, 1.27)  *P*=0.16 | 0.81 (0.69, 0.95)  *P*=0.01 | 0.79 (0.70, 0.88)  *P* < .001 |
|  | Model 2 | 1.26 (1.05, 1.52)  *P*=0.012 | 1.15 (1.00, 1.32)  *P*=0.05 | 0.83 (0.72, 0.97)  *P*=0.02 | 0.80 (0.71, 0.90)  *P* < .001 |
|  | Model 3 | 1.18 (0.99, 1.40)  *P*=0.60 | 1.08 (0.95, 1.24)  *P*=0.24 | 0.80 (0.79, 0.94)  *P*=0.01 | 0.78 (0.70, 0.88)  *P* < .001 |
| Happening Soon  IRR (95% CI) | Model 1 | 1.38 (1.00, 1.92)  *P*=0.052 | 1.16 (0.90, 1.50)  *P*=0.26 | 0.71(0.56, 0.91)  *P*=0.01 | 0.68 (0.56, 0.82)  *P* < .001 |
|  | Model 2 | 1.53 (1.13, 2.08)  *P*=0.01 | 1.30 (1.02, 1.67)  *P*=0.04 | 0.87 (0.67, 1.09)  *P*=0.23 | 0.77 (0.63, 0.93)  *P*=0.01 |
|  | Model 3 | 1.31 (0.95, 1.79)  *P*=0.10 | 1.11 (0.87, 1.42)  *P*=0.41 | 0.72 (0.56, 0.92)  *P*=0.01 | 0.68 (0.56, 0.82)  *P* < .001 |
| I Would Like  IRR (95% CI) | Model 1 | 1.59 (0.89, 2.84)  *P*=0.12 | 1.47 (0.83, 2.61)  *P*=0.19 | 0.96 (0.67, 1.37)  *P*=0.84 | 0.97 (0.68, 1.38)  *P*=0.87 |
|  | Model 2 | 1.55 (0.85, 2.82)  *P*=0.16 | 1.46 (0.79, 2.70)  *P*=0.23 | - 1. (0.68, 1.50)   2. *P*=0.95 | 0.98 (0.68, 1.43)  *P*=0.932 |
|  | Model 3 | 1.52 (0.87, 2.64)  *P*=0.14 | 1.40 (0.80, 2.43)  *P*=0.24 | 0.97 (0.67, 1.38)  *P*=0.85 | 0.98 (0.69, 1.39)  *P*=0.90 |
| Messages  IRR (95% CI) | Model 1 | 1.45 (1.15, 1.83)  *P*=0.002 | 1.33 (1.06, 1.67)  *P*=0.02 | 0.67 (0.55, 0.83)  *P* < .001 | 0.65 (0.53, 0.78)  *P* < .001 |
|  | Model 2 | 1.52 (1.20, 1.93)  *P* =0.001 | 1.41(1.12, 1.78)  *P*=0.003 | 0.71 (0.58, 0.87)  *P* =0.001 | 0.67 (0.55, 0.82)  *P* < .001 |
|  | Model 3 | 1.37 (1.09, 1.72)  *P*=0.01 | 1.26 (1.01, 1.57)  *P*=0.04 | 0.67 (0.55, 0.83)  *P* < .001 | 0.65 (0.53, 0.78)  *P* < .001 |
| My Health  IRR (95% CI) | Model 1 | 0.89 (0.55, 1.42)  *P*=0.61 | 0.93 (0.66, 1.32)  *P*=0.69 | 0.44 (0.30, 0.65)  *P* < .001 | 0.51 (0.36, 0.73)  *P* < .001 |
|  | Model 2 | 1.06 (0.68, 1.66)  *P*=0.79 | 0.96 (0.67, 1.39)  *P*=0.84 | 0.52 (0.36, 0.77)  *P*=0.001 | 0.51 (0.36, 0.74)  *P* < .001 |
|  | Model 3 | 0.85 (0.58, 1.33)  *P*=0.47 | 0.91 (0.65, 1.27)  *P*=0.57 | 0.44 (0.30, 0.66)  *P* < .001 | 0.50 (0.35, 0.73)  *P* < .001 |
| Notes  IRR (95% CI) | Model 1 | 0.81 (0.30, 2.23)  *P*=0.69 | 0.63 (0.24, 1.71)  *P*=0.36 | 0.58 (0.25, 1.32)  *P*=0.19 | 0.54 (0.24, 1.24)  *P*=0.15 |
|  | Model 2 | 0.76 (0.27, 2.18)  *P*=0.62 | 0.67 (0.23, 2.01)  *P*=0.48 | 0.63 (0.28, 1.46)  *P*=0.28 | 0.63 (0.28, 1.45)  *P*=0.28 |
|  | Model 3 | 0.72 (0.26, 1.99)  *P*=0.53 | 0.55 (0.20, 1.50)  *P*=0.25 | 0.60 (0.26, 1.37)  *P*=0.23 | 0.57 (0.25, 1.31)  *P*=0.19 |
| Taking Care of Me  IRR (95% CI) | Model 1 | 1.22 (0.99, 1.51)  *P*=0.07 | 1.09 (0.90, 1.33)  *P*=0.35 | 0.65 (0.54, 0.79)  *P* < .001 | 0.64 (0.54, 0.75)  *P* < .001 |
|  | Model 2 | 1.29 (1.05, 1.60)  *P*=0.02 | 1.16 (0.97,1.39)  *P*=0.11 | 0.68 (0.57, 0.82)  *P* < .001 | 0.66 (0.56, 0.77)  *P* < .001 |
|  | Model 3 | 1.21 (0.98, 1.49)  *P*=0.07 | 1.10 (0.91,1.34)  *P*=0.31 | 0.63 (0.52, 0.76)  *P* < .001 | 0.62 (0.52, 0.73)  *P* < .001 |
| To Learn  IRR (95% CI) | Model 1 | 1.18 (0.84, 1.64)  *P*=0.34 | 1.14 (0.81, 1.59)  *P*=0.45 | 0.74 (0.58, 0.95)  *P*=0.02 | 0.75 (0.58, 0.95)  *P*=0.02 |
|  | Model 2 | 1.29 (0.92, 1.82)  *P*=0.15 | 1.25 (0.90, 1.74)  *P*=0.19 | 0.79 (0.61, 1.01)  *P*=0.06 | 0.78 (0.61, 1.01)  *P*=0.06 |
|  | Model 3 | 1.13 (0.82, 1.56)  *P*=0.45 | 1.10 (0.80, 1.52)  *P*=0.56 | 0.74 (0.58, 0.95)  *P*=0.02 | 0.75 (0.58, 0.96)  *P*=0.02 |
| Tutorial  IRR (95% CI) | Model 1 | 1.11 (0.99, 1.25)  *P*=0.102 | 1.12 (0.99, 1.26)  *P*=0.07 | 0.89 (0.81, 0.98)  *P*=0.02 | 0.92 (0.84,1.00)  *P*=0.05 |
|  | Model 2 | 1.14 (1.01, 1.28)  *P*=0.03 | 1.15 (1.03, 1.29)  *P*=0.02 | 0.91 (0.83, 0.99)  *P*=0.04 | 0.93 (0.86, 1.01)  *P*=0.10 |
|  | Model 3 | 1.09 (0.98, 1.23)  *P*=0.15 | 1.091 (0.97, 1.22)  *P*=0.15 | 0.89 (0.80, 0.98)  *P*=0.02 | 0.92 (0.84, 1.00)  *P*=0.054 |
| Comprehensive user^d^  OR (95% CI) | Model 1 | 1.09 (0.79, 1.50)  *P*=0.60 | 1.02 (0.73, 1.42)  *P*=0.90 | 0.56 (0.44, 0.71)  *P* < .001 | 0.57 (0.45, 0.73)  *P* < .001 |
|  | Model 2 | 1.52 (1.19, 1.94)  *P*=0.001 | 1.44 (1.12, 1.85)  *P*=0.01 | 0.70 (0.58, 0.84)  *P* < .001 | 0.62 (0.48, 0.80)  *P* < .001 |
|  | Model 3 | 1.06 (0.78, 1.44)  *P*=0.72) | 0.99 (0.72, 1.37)  *P*=0.97 | 0.55(0.43, 0.70)  *P* < .001 | 0.56 (0.44, 0.72)  *P* < .001 |
| Composite user^e^  OR (95% CI) | Model 1 | 1.26 (0.88, 1.83)  *P*=0.21 | 1.15 (0.77, 1.72)  *P*=0.50 | 0.45 (0.33, 0.62)  *P* < .001 | 0.42(0.29, 0.60)  *P* < .001 |
|  | Model 2 | 1.51 (1.03, 2.22)  *P*=0.03 | 1.34 (0.89, 2.01)  *P*=0.16 | 0.57 (0.41, 0.79)  *P*=0.001 | 0.53 (0.39, 0.77)  *P* < .001 |
|  | Model 3 | 1.20 (0.84, 1.72)  *P*=0.31 | 1.08 (0.73, 1.60)  *P*=0.68 | 0.44 (0.32, 0.61)  *P* < .001 | 0.41 (0.29, 0.59)  *P* < .001 |

*Notes:* Models adjusted for age at enrollment, gender, race, Charlson Comorbidity Index, and length of provisioning time of the inpatient tablet.

^a^ Model 1: MCB use from up to three admissions inclusive of the study enrollment admission. MC user status defined as Prior Users (n=695) were patients with any past recorded MC use prior to their enrollment admission; New Users (n=214) who first used MC during their enrollment admission or within 90 days of their enrollment into the trial; and Non-Users (n=662) who did not use MC either before, during, or after their enrollment admission or those who first used MC 90 days after enrollment into the trial.

^b^ Model 2: MCB use from the study enrollment admission. MC user status defined as Prior Users (n=695) were patients with any past recorded MC use prior to their enrollment admission; New Users (n=214) who first used MC during their enrollment admission or within 90 days of their enrollment into the trial; and Non- Users (n=662) who did not use MC either before, during, or after their enrollment admission or those who first used MC 90 days after enrollment into the trial.

^c^ Model 3: MCB use from up to three admissions inclusive of the study enrollment admission. MC user status defined as Prior Users (n=695) were patients with any past recorded MC use prior to their enrollment admission; New Users (n=240) who first used MC during their enrollment admission or within 180 days of their enrollment into the trial; and Non-Users (n=636) who did not use MC either before, during, or after their enrollment admission or those who first used MC 180 days after enrollment into the trial.

^d^ Comprehensive user defined as use of eight or more MCB functions.

^e^ Composite user in Model 1 defined as a comprehensive user and high-frequency user of MCB. High-frequency use defined as having total number of MCB sessions greater than or equal the 75^th^ percentile (41 sessions).

Composite user of MCB in Model 2 defined as a comprehensive user and high-frequency user of MCB. High-frequency use defined as having total number of MCB sessions greater than or equal the 75^th^ percentile (32 sessions).

Composite user in Model 3 defined as a comprehensive user and high user of MCB. High-frequency use defined as having total number of MCB sessions greater than or equal the 75^th^ percentile (33 sessions).

IRR, incidence rate ratio; OR, odds ratio; CI, confidence interval.
